# Population Based Model of Human Embryonic Stem Cell (hESC) Differentiation during Endoderm Induction

**DOI:** 10.1371/journal.pone.0032975

**Published:** 2012-03-12

**Authors:** Keith Task, Maria Jaramillo, Ipsita Banerjee

**Affiliations:** 1 Department of Chemical Engineering, University of Pittsburgh, Pittsburgh, Pennsylvania, United States of America; 2 Department of Bioengineering, University of Pittsburgh, Pittsburgh, Pennsylvania, United States of America; University of Udine, Italy

## Abstract

The mechanisms by which human embryonic stem cells (hESC) differentiate to endodermal lineage have not been extensively studied. Mathematical models can aid in the identification of mechanistic information. In this work we use a population-based modeling approach to understand the mechanism of endoderm induction in hESC, performed experimentally with exposure to Activin A and Activin A supplemented with growth factors (basic fibroblast growth factor (FGF2) and bone morphogenetic protein 4 (BMP4)). The differentiating cell population is analyzed daily for cellular growth, cell death, and expression of the endoderm proteins Sox17 and CXCR4. The stochastic model starts with a population of undifferentiated cells, wherefrom it evolves in time by assigning each cell a propensity to proliferate, die and differentiate using certain user defined rules. Twelve alternate mechanisms which might describe the observed dynamics were simulated, and an ensemble parameter estimation was performed on each mechanism. A comparison of the quality of agreement of experimental data with simulations for several competing mechanisms led to the identification of one which adequately describes the observed dynamics under both induction conditions. The results indicate that hESC commitment to endoderm occurs through an intermediate mesendoderm germ layer which further differentiates into mesoderm and endoderm, and that during induction proliferation of the endoderm germ layer is promoted. Furthermore, our model suggests that CXCR4 is expressed in mesendoderm and endoderm, but is not expressed in mesoderm. Comparison between the two induction conditions indicates that supplementing FGF2 and BMP4 to Activin A enhances the kinetics of differentiation than Activin A alone. This mechanistic information can aid in the derivation of functional, mature cells from their progenitors. While applied to initial endoderm commitment of hESC, the model is general enough to be applicable either to a system of adult stem cells or later stages of ESC differentiation.

## Introduction

Embryonic stem cells (ESC) have gained much attention in recent years for their ability to differentiate into cells of any of the three primary germ layers (endoderm, mesoderm, and ectoderm) as well as to remain in a pluripotent state under appropriate conditions [1], [2]. Numerous types of endoderm-like cells emerging during gastrulation have been described, including primitive, visceral, parietal, and definitive endoderm [3]. The definitive endoderm, henceforth referred to as endoderm, gives rise to such tissues as liver and pancreas [4], [5]. A focus of stem cell study in recent years has been the directed differentiation of ESC into endoderm cells for subsequent differentiation into hepatic or pancreatic lineage. Extensive research has established the possibility of deriving endoderm following alternate routes. What is however lacking is a thorough mechanistic investigation of the dynamics of differentiation. For example, studies have shown Nodal, a key component in the Nodal signaling pathway which induces endoderm, can be mimicked *in vitro* by human Activin A [6], [7]. Endoderm formation with the addition of Activin A has been experimentally verified in numerous studies [8]–[10]. However, the routes by which pluripotent cells differentiate to the endoderm germ layer during Activin A exposure have been less studied. Furthermore, while there has been some interest in modeling the gene regulatory network of differentiating stem cells using population averaged information [11], [12], the heterogeneity and stochasticity of the process of differentiation demands more careful analysis. Mechanistic studies will be beneficial in efficient derivation of mature, functional cell types, and mathematical models which incorporate differences at the cellular level can be used to elucidate these mechanisms.

The current study aims to gain a better understanding of endoderm differentiation of human embryonic stem cells (hESC) through the development of a population based model. We base our model on earlier reports [13], [14] which model hematopoietic stem cell differentiation and organization, and modify it for the system of embryonic stem cells. Overall, the model involves a stochastic simulation, where a population of cells is evolved following specific user defined rules through which the system dynamics can be extracted. The model predicts three aspects of endoderm formation: total cell proliferation, cell death, and lineage commitment. In order to understand the mechanism favoring the process of stem cell differentiation we simulate several alternate mechanisms and compare the simulated dynamics with our experimental data. Endoderm is experimentally induced in hESC through alternate pathways: addition of Activin A and Activin A supplemented with the growth factors basic fibroblast growth factor (FGF2) and bone morphogenetic protein 4 (BMP4) [9], [15], [16]. Differentiation dynamics of the cell population is experimentally tracked by analyzing percentage of cell population expressing endoderm specific proteins: Sox17 and CXCR4 [17], [18]. A single mechanism thought to be involved in endoderm differentiation was identified by agreement between the experimental data and the simulated dynamics using this mechanism. Through this integrated experimental and mathematical approach, we shed light on how hESC differentiate during endoderm induction.

## Methods

### Cell Culture and Endoderm Induction

Human embryonic stem cells (H1 cell line) were cultured under feeder-free condition. 6-well culture dishes were incubated with Matrigel™ coating (hESC-qualified Matrix, BD Biosciences, San Jose, CA, USA) for 30 minutes. hESC colonies (p93) were plated onto the Matrigel layer with 1 mL mTeSR®1 hESC media and supplement (Stem Cell Technologies, Vancouver, BC, Canada). The cells were incubated at 37°C in 5% CO_2_, and the mTeSR®1 media was replaced daily. For the differentiation study, the current work chose to compare two conditions to induce endoderm: human Activin A (henceforth referred to as ‘Condition A’) and human Activin A supplemented with the growth factors FGF2 and BMP4 (‘Condition B’). To commence endoderm induction, DMEM/F12 (Invitrogen, Carlsbad, CA, USA), 1×B27® Supplement (Invitrogen), and 0.2% Bovine Serum Albumin (BSA, Sigma-Aldrich,St. Louis, MO, USA) supplemented with 100 ng/mL human Activin A (Condition A; R&D Systems, Minneapolis, MN, USA) or 100 ng/mL human Activin A, 100 ng/mL FGF2 (Invitrogen), and 100 ng/mL BMP4 (Condition B; R&D Systems) was used as differentiation media, which was replaced daily for a total of five days. Upon induction of differentiation cells were harvested on a daily basis for subsequent analysis. For each well, the supernatant was collected, the plated cells were dissociated with Trypsin+EDTA (Invitrogen), Trypan Blue (Sigma-Aldrich) was added to distinguish live from dead , and the cells were counted using a standard hemacytometer,

### Flow Cytometry

For flow cytometry, harvested cells were first fixed for 15 minutes in 4% methanol-free formaldehyde (Thermo Scientific, Rockford, IL, USA) in phosphate-buffered saline (PBS). Cells were washed twice and permeabilized in 0.1% Saponin (Sigma-Aldrich)+0.5% BSA in PBS for 30 minutes. To block non-specific binding, the cells were incubated in 3% BSA+0.25% dimethyl sulfoxide (DMSO, Fisher Scientific, Hampton, NH, USA)+0.1% Saponin in PBS for 30 minutes. A portion of cells were then set aside as the negative control (secondary antibody only without primary). The cells to be used as the positive samples were then incubated in blocking buffer with goat anti-human sox17 (R&D Systems) and rabbit anti-human cxcr4 (Abcam, Cambridge, MA, USA) primary antibodies, 1∶200 dilution, for 30 minutes. The cells were washed twice with blocking buffer, resuspended in the buffer, and incubated with donkey anti-goat APC (1∶350 dilution; Santa Cruz Biotechnology, Santa Cruz, CA, USA) and donkey anti-rabbit FITC (1∶200 dilution; Santa Cruz Biotechnology) for 30 minutes (both the samples and negative control). Two washings were followed by 10 minutes of 0.2% tween-20 (Bio-Rad, Hercules, CA, USA) to further eliminate non-specific staining. Cells were washed and transferred to flow cytometry tubes. Accuri C6 © Flow Cytometer was used to quantify sox17-APC and cxcr4-FITC expression. Cells stained with the secondary antibody only (without primary antibody) were first analyzed; this population was taken as the negative, and the gate was set beyond these cells to eliminate false positives due to auto-fluorescence and non-specific secondary antibody binding. The completely stained samples (primary and secondary antibody stained) were then analyzed, and the percentage of the population falling within the set gate was recorded as the positive sample for the respective antibody.

### Quantitative Polymerase Chain Reaction

To quantify mRNA levels, harvested cells were lysed and mRNA was extracted and purified using a Nucleospin II RNA kit (Macherey-Nagel, Bethlehem, PA, USA). The RNA quantity and quality was measured using a SmartSpec™ Plus spectrophotometer (Bio-Rad), after which reverse transcription was performed with the ImProm II Reverse Transcriptase System (Promega, Madison, WI, USA). cDNA levels of Gapdh (left primer: 5′ acg acc act ttg tca agc tca ttt c 3′; right primer: 5′ gca gtg agg gtc tct ctc ttc ctc t 3′), Oct4 (left primer: 5′ ctg ggt tga tcc tcg gac ct 3′; right primer: 5′ cac aga act cat acg gcg gg 3′), and Brachyury (left primer: 5′ tgc ttc cct gag acc cag tt 3′; right primer: 5′ gat cac ttc ttt cct ttg cat caa g 3′) were measured with quantitative polymerase chain reaction (qPCR) using an Mx3005P system (Agilent, Santa Clara, CA, USA) and Brilliant SYBR Green qPCR Master Mix (Agilent).

### Mathematical Model

The system of stem cell differentiation to endoderm is modeled using a stochastic population-based model. The basic formulation of the model is based on earlier reports for hematopoietic stem cells [13], [14]. Here we are introducing some modifications to adapt it to the embryonic stem cell system, followed by a stringent model analysis using parameter sensitivity and feasibility studies. In this section we briefly summarize the working principle of the model along with our modifications. We refer the readers to [13], [14] for details of model specifics and to Figure S1 for the pseudo-code describing the implementation of the main simulation, parameter ensemble, and sensitivity analysis.

#### Signaling Regimes

The model is initiated by a population of cells, the properties of which are evolved by specific pre-assigned rules. The cells are primarily categorized into two signaling regimes: Ω and A; Ω can be considered as an active regime supporting cellular proliferation and differentiation, while A is a more dormant regime where cells are quiescent and prone to dedifferentiation. The cells can transfer in between these two regimes, an event decided upon primarily by a cell-specific parameter, termed as ‘a’ value. This parameter ‘a’ is randomly assigned to each cell at the beginning of simulation, and is updated at each time step. The probability of transfer to/from a regime is dependent on this parameter along with the number of cells in the destination regime. This ‘a’ value is unaltered in the A regime while it progressively reduces in the Ω regime, and when it falls below a threshold the cells lose their ability to transfer to the A regime.

#### Proliferation, Cell Death, and Differentiation Rules

Each cell is randomly assigned a maximum life span, exceeding which it will die. While the cells age in the Ω regime, they neither proliferate nor age in the A regime. Proliferation is allowed in the Ω regime for an amount of time which is cell dependent, after which the cell enters a senescent stage and will not proliferate. Furthermore, an individual cell is allowed to proliferate only after it loses the capability to pass into A regime having crossed the ‘a’ threshold value.

Cellular differentiation is governed by the ‘lineage propensity’ parameter, representing a cell's likelihood to differentiate into a particular lineage. Only the Ω regime allows an increase in lineage propensity. While updating the propensity of differentiation to a particular lineage, all the possible lineages are competing and any can be updated, with the one with higher propensity having a higher probability of being selected. Once a cell's propensity exceeds a threshold level, the cell is considered committed to that particular lineage and will retain its differentiated phenotype. If this threshold has not been exceeded and if the cell is chosen to be transferred to the A regime, the propensity values will converge to an average value. The model is therefore able to track specific germ layer populations, and through this the percent of the population positive for Sox17 (visceral and definitive endoderm marker) and CXCR4 (definitive endoderm and mesendoderm marker) [17]–[19] can be extracted.

#### Mechanism of hESC Differentiation

The current work is focused on the mechanistic investigation of the dynamics of hESC induction into endoderm. Using the platform of the stochastic population based model we investigated several alternate mechanisms and analyzed them for agreement with experimental data. Three characteristics of the differentiation process were chosen to be investigated: the presence/absence of an intermediate germ layer, mesendoderm, which subsequently gives rise to mesoderm and endoderm [20]; the presence/absence of CXCR4, in mesoderm ; and whether proliferation of a specific differentiated cell phenotype is favored over others. Combination of aforementioned attributes results in 12 alternate mechanisms, each of which were incorporated into the model and analyzed for agreement with experimental data. It is expected that the most likely mechanism will best describe the experimental dynamics of the stem cell system.

The incorporation of mesendoderm involved a two stage differentiation scheme. In the first stage, hESC are able to differentiate into either mesendoderm or visceral endoderm. Once cells are committed to the mesendoderm lineage, several of their attributes are re-initialized, such as their ‘a’ value and lineage propensities. The mesendodermal cells can then further differentiate into endoderm or mesoderm. Differences in the proliferation potential of different phenotypes were incorporated by considering 3 scenarios: proliferation of hESC and mesendoderm; proliferation of hESC, mesendoderm and endoderm; and proliferation of all phenotypes.

#### Convergence Study

As with any stochastic model, the number of model runs necessary to obtain a converged solution needs to be determined. An additional parameter of the current model is the initial cell population, which affects the solution over a certain range. A two-dimensional convergence study was undertaken, wherein the effects of both stochastic run number and initial cell population on model output were determined. The convergence test allows determination of the minimum number of stochastic runs and initial cell population beyond which the model output does not significantly change. All results reported here are using the converged parameter values.

#### Sensitivity Analysis

Sensitivity analysis was performed to determine the relative importance of parameters in affecting the outputs of cellular growth, death, and lineage commitment. Because this model is probabilistic in nature, traditional ways of determining local sensitivity, e.g. partial derivative of output with respect to an input, cannot be employed. A stochastic analysis was therefore chosen, using differences in output histograms of nominal and perturbed parameters, S, to estimate parameter sensitivity [21] as:
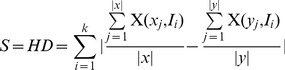
(1)where *x_j_* and *y_j_* are the individual elements in the nominal and perturbed histograms, respectively, *I_i_* represents the range of each bin *i* in the nominal histogram, X is a counting variable which takes on a value of 1 if *x_j/_y_j_* is within the interval *I_i_*, |x| and |y| are cardinalities of data sets x (nominal) and y (perturbed), respectively, and *k* is the number of bins, which was determined by calculating the appropriate bin size of the nominal output histogram by the Freedman-Daconis rule [22]:
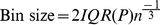
(2)Where IQR(P) is the interquartile range of a sample population *P* and *n* is the number of observations in *P*. The number of bins was different for each output, and ranged from 37 to 51.

For sensitivity analysis, each parameter was perturbed by 10% while keeping the rest at the nominal value. For each bin in the nominal output histogram, the difference between the percentage of total nominal histogram elements residing in that bin and the percentage of the total perturbed histogram elements residing in that bin is calculated. The sum of the absolute value of this difference over all of the bins is the histogram distance.

#### Parameter Ensemble

Having determined the most sensitive model parameters, the next step is to determine an appropriate value of the parameters which best estimates the experimental data. We realize that a single parameter combination may not be adequate in describing the experimental data; instead, there exists a parameter hyper-space adequately satisfying the data. Hence an ensemble parameter estimation was performed by randomly generating initial guesses from the hyper-space of the sensitive parameters. The model was simulated with 10000 random parameter samples; for each of these simulations the least square estimate is determined between experimental data and model output. These simulations were run for each mechanism and condition under investigation, and parameter ensembles were generated by considering only those parameter sets which meet certain error constraints. Following the above detailed methodology we evaluated the model predictions obtained from the different mechanisms and compared them with experimental data to determine the most plausible mechanism.

## Results

### Experimental Data

**Figure 1:**
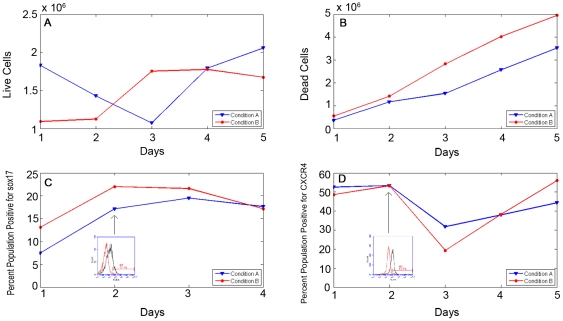
Experimental results of cell behavior during endoderm induction. Cellular growth (A) and death (B) dynamics for Conditions A and B. Temporal behavior of cellular population positive for Sox17 (C) and CXCR4 (D). Inset: Representative output of flow cytometry data. Red histogram: secondary antibody only control. Black histogram: sample. Red gate denotes sample taken to be positive.

The hESC culture was analyzed for cellular growth and death dynamics during endoderm induction by both Activin A (Condition A) and Activin A/FGF2/BMP4 (Condition B) conditions as illustrated in Figure 1. Cellular growth kinetics was found to exhibit non-linear dynamics, while cell death remained predominantly linear over time. A proliferation lag time is exhibited in Condition A up until Day 3, during which time the number of live cells decreases because of cell death. Beyond this time, cells begin to proliferate in a roughly linear fashion. Interestingly, the majority of cell growth in Condition B occurs before Day 3.

The differentiated cell population was analyzed by flow cytometry for percent of cells positive for Sox17 and CXCR4 for each day of differentiation. Figure 1 represents the dynamics of Sox17 (C) and CXCR4 (D) expression for both the experimental conditions, with the insets illustrating representative flow cytometry results. Details of the flow data are presented in Figure S2. The population positive for Sox17 exhibits quadratic behavior with a maximum percentage of ∼19–23% at ∼2–3 days (depending on the condition). The fraction of cells positive for CXCR4 is relatively constant until the second day, after which there is a significant drop. Subsequently, there is an approximately linear increase in the CXCR4 population which is more prominent in Condition B.

### Mathematical Model

#### Model Parameter Analysis

**Figure 2:**
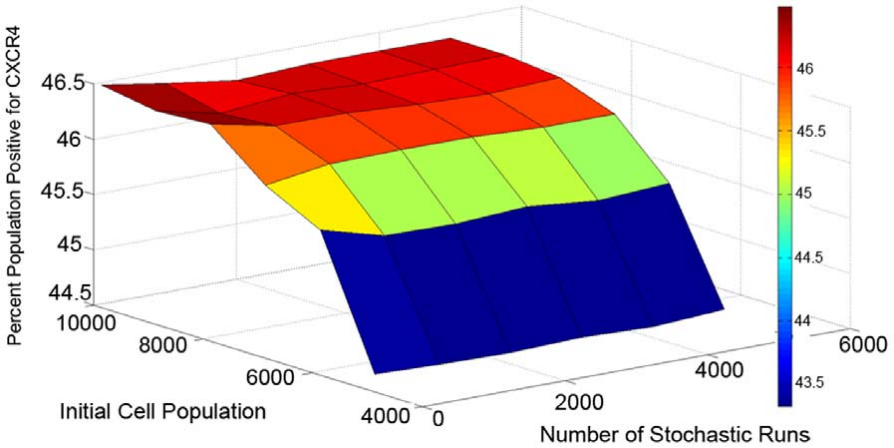
Convergence study of simulated cell population over various initial cell populations and total stochastic runs. Output is percent of the simulated population positive for CXCR4, averaged over all stochastic runs at Day 5.

In the next step the developed stochastic model was used to test the proposed mechanisms for agreement with experimental data. The mathematical model involves multiple parameters which require detailed analysis before the model can be used for prediction. The parameters can be grouped into two categories: (a) simulation parameters which affect the convergence behavior of the simulation and (b) model parameters which affect specific model output for the converged simulation. Two parameters were identified to be simulation parameters: initial cell population and number of stochastic runs. These parameter values were optimized by performing a two-dimensional convergence study, as illustrated in Figure 2 for the CXCR4-positive population output. Overall it is observed that the initial cell population has a more dominant effect on convergence, while the effect of stochastic runs was rather weak beyond 2000 runs. Following this analysis an initial cell population of 9000 and total stochastic runs of 4000 was used for subsequent simulations.

**Figure 3:**
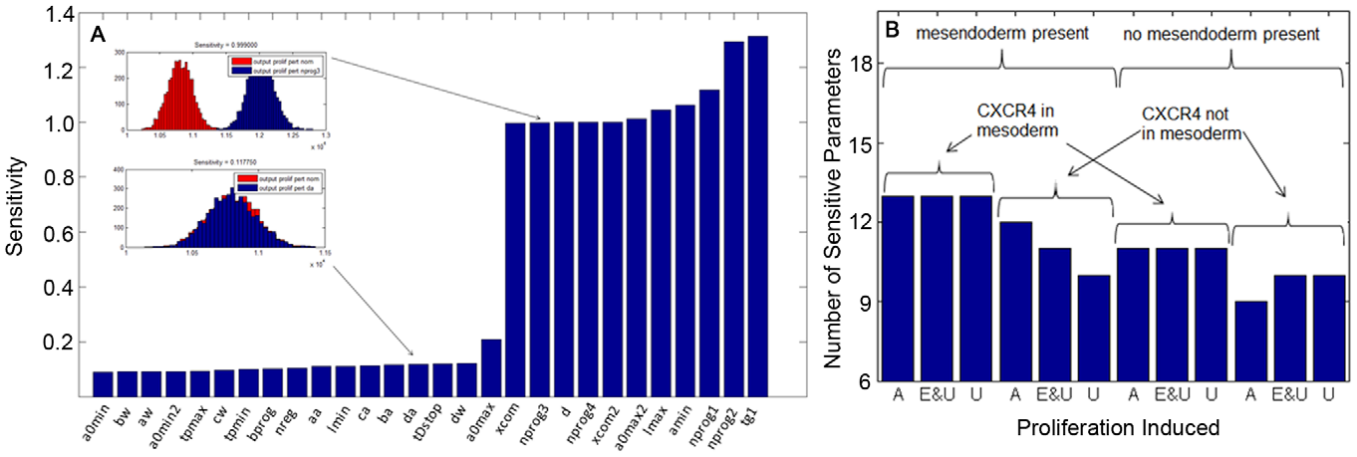
Sensitivity analysis of population based model. (A) Cellular growth sensitivity to each of the parameters, perturbed by 10%. Parameter definitions listed in Table S1. (inset) Comparison of cellular growth output histogram from nominal (red) and perturbed (blue) parameters. (B) Number of sensitive parameters determined for each mechanistic model. Proliferation induced: A, all phenotypes; E&U, endoderm and uncommitted (hESC and mesendoderm); U, uncommitted only.

A detailed sensitivity analysis was performed for the model parameters in order to determine the relative importance of the parameters in determining model output. As detailed in Equation 1, the measure of histogram distance is used to represent the parameter sensitivity associated with a specific model output. Figure 3A illustrates the model parameter sensitivity to the output of cellular growth, as concluded from the shift in histogram distance (inset). A clear jump in the sensitivity is observed, with a large difference between parameters with low and high sensitivity.

While Figure 3A represents the parameter sensitivity to model output of cellular growth, similar analysis was also performed for all of the model outputs (data not shown). Overall it was observed that even though the magnitude of sensitivity differs between outputs, the highly sensitive parameters were mostly conserved across outputs. Furthermore, in the present study we are investigating multiple competing mechanisms, which require modification of the model formulation. Since the effect of such modifications on the parameter sensitivity is not intuitively obvious, similar analysis was repeated for each of the 12 proposed mechanisms. Figure 3B summarizes the number of sensitive parameters for each of the mechanisms. From the analysis, eight classes of parameters were consistently observed to have the highest sensitivity:

- a_min_: ‘a’ value threshold beyond which a cell enters the proliferation phase
- a_0max_: The initial cell population is randomly assigned an ‘a’ value, with an upper limit of a_0max_
- x_com_: lineage propensity threshold beyond which a cell is committed to a particular lineage
- d: factor with which ‘a’ decreases
- t_g1_: time cell stays in G1 phase of cell cycle. Only in this phase can a cell differentiate and transfer to the A regime from the Ω regime
- l_max_: upper value of range of cell population's life span
- nprog_i_: factor in determining magnitude of propensity updates for each lineage i
- aa: parameter in determining the probability of a cell transferring from the Ω regime to the A regime

#### Ensemble Parameter Estimation

**Figure 4:**
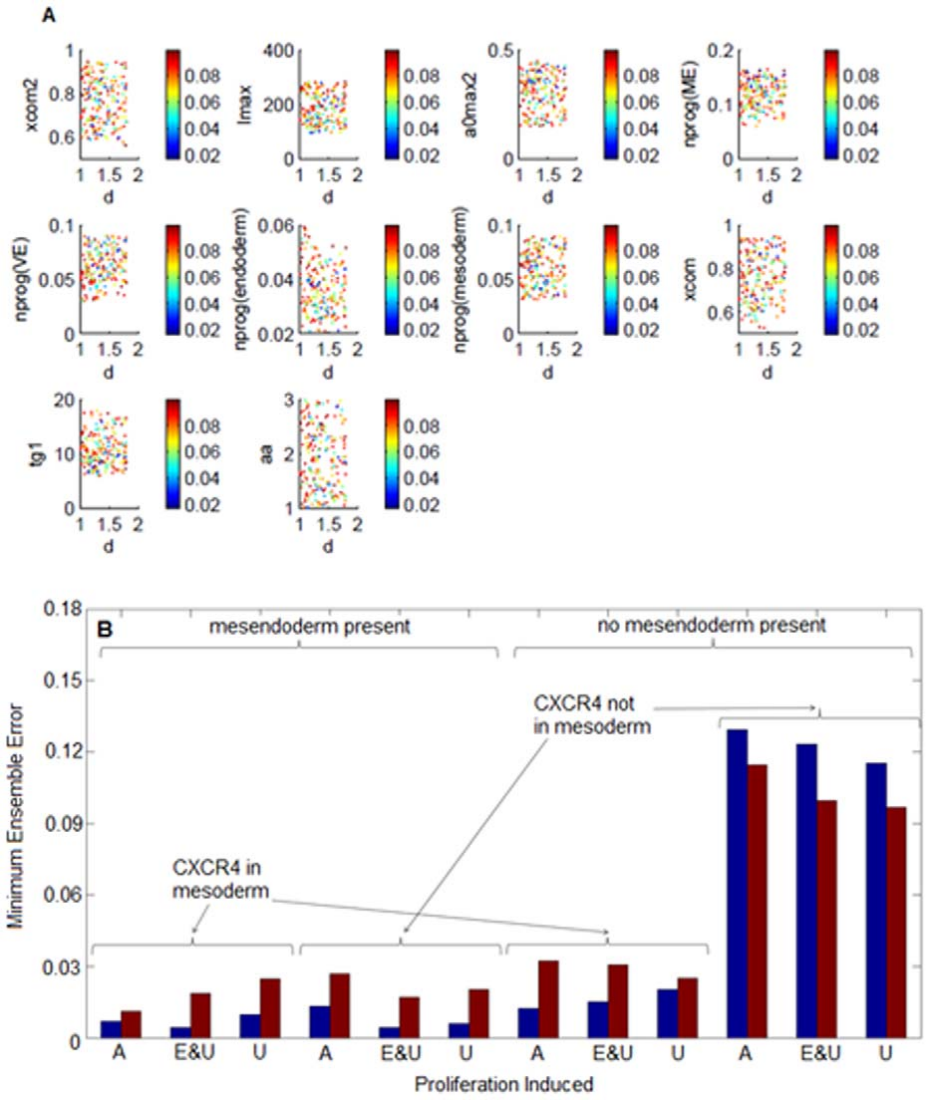
Ensemble parameter estimation and model errors. (A) Parameter values for Mechanism B ensemble yielding errors of less than 0.025. Each parameter is compared to the most sensitive parameter, ‘d’. Color bar denotes the ensemble error for that particular parameter value. (B) Minimum ensemble error generated for each mechanistic model. Proliferation induced: A, all phenotypes; E&U, endoderm and uncommitted (hESC and mesendoderm); U, uncommitted only. Blue, Condition A; Red, Condition B.

Having determined the sensitive parameters for each of the mechanisms, the next step is to determine the optimum parameter values which will result in best agreement with experimental data. In literature, biological samples are typically defined as being ‘sloppy’ [23] with a broad ensemble of parameters satisfying the error constraints. Accordingly we also target identification of representative ensemble of parameters. The model is formulated to capture the dynamics of cellular growth, death and differentiation, the output of differentiation being of most interest. Hence the model parameters were optimized with respect to differentiation dynamics, while growth kinetics and the dynamics of cell death were used for verification. A projection of the simulated error onto a 2-dimensional parameter space (for the mechanism which incorporates mesendoderm and promotes proliferation of both uncommitted and endodermal cells without CXCR4 being expressed in mesoderm (‘Mechanism B’)) is shown in Figure 4A. Although it was initially thought that a trend might be observed between the error and values of the parameter ensemble, Figure 4A shows that there is a lack of any correlation between multiple parameters and associated errors (shown for parameter ‘d’; further analysis of all parameter combinations yielded same results, data not shown). Figure 4B illustrates the minimum ensemble error for each of the proposed mechanisms simulated under the two endoderm induction conditions, the error being evaluated according to least square formulation.

#### Mechanism Evaluation: Endoderm Induction by Activin A

**Figure 5:**
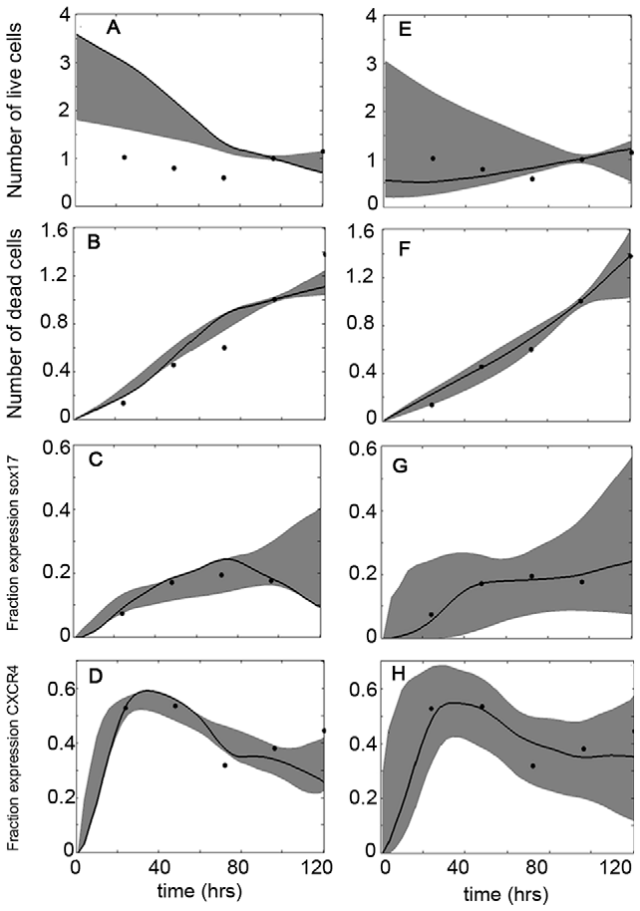
Simulated output dynamics compared to experimental data (Condition A). Grey band denotes the ensemble of simulations having an error less than the threshold, with the single solid black curve showing the best fit. Black circles represent the experimental data points. (A–D): Growth kinetics, cell death, fraction of population positive for Sox17 and CXCR4 dynamics, respectively, of Mechanism A; error threshold of 0.05. (E–F) Growth kinetics, cell death, fraction of population positive for Sox17 and CXCR4 dynamics, respectively, of Mechanism B; error threshold of 0.025. Cellular growth and death normalized to Day 4.

As shown in Figure 4B, the absence of mesendoderm gives rise to large errors, in some cases an order of magnitude higher than their counterpart models which include mesendoderm. If one considers the Activin A only condition, the most accurate mechanisms include those which incorporate mesendoderm and promote proliferation of both uncommitted and endoderm germ layer both with (‘Mechanism A’) and without (‘Mechanism B’) CXCR4 in mesoderm. Since Figure 4B illustrates the accuracy of the model in predicting differentiation dynamics only, the performance of the 2 prospective mechanisms were further verified with the help of growth kinetics and the dynamics of cell death. Figure 5 illustrates the ensemble simulation of all the model outputs and its comparison with experimental data. While both the mechanisms had excellent performance in predicting Sox17 and CXCR4 dynamics (Figure 5 C,D,G,H) they differed significantly in predicting growth kinetics and cell death dynamics (Figure 5 A,B,E,F). Figure 5 clearly illustrates that Mechanism B performs better in describing both growth kinetics and cell death dynamics compared to Mechanism A. Hence the former was chosen to be the most likely mechanism for Condition A.

#### Mechanism Evaluation: Endoderm Induction by Activin A Supplemented by Growth Factors

**Figure 6:**
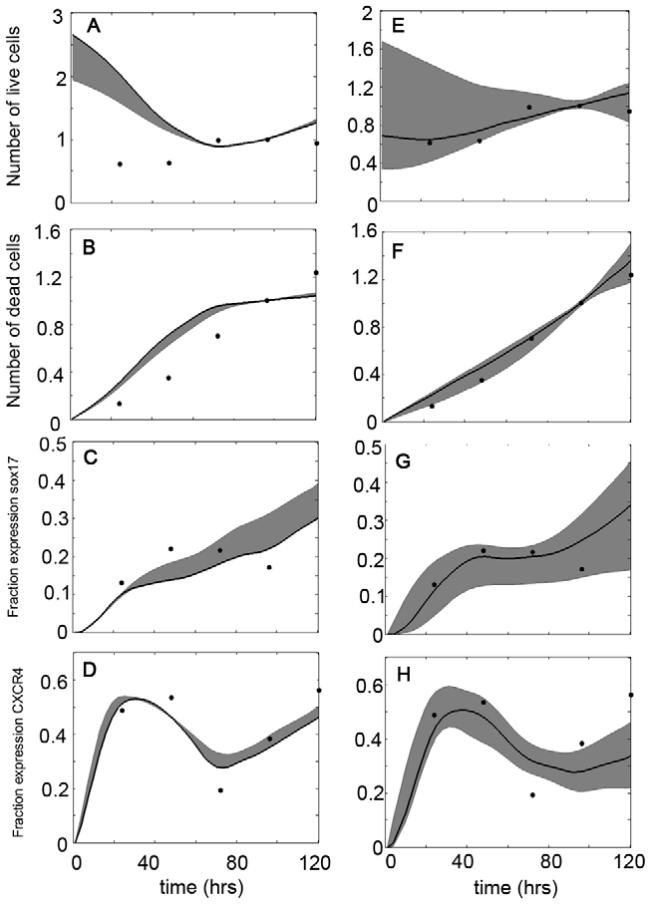
Simulated output dynamics compared to experimental data (Condition B). Grey band denotes the ensemble of simulations having an error less than the threshold, with the single solid black curve showing the best fit. Black circles represent the experimental data points. (A–D): Growth kinetics, cell death, fraction of population positive for Sox17 and CXCR4 dynamics, respectively, of Mechanism C; error threshold of 0.1. (E–F) Growth kinetics, cell death, fraction of population positive for Sox17 and CXCR4 dynamics, respectively, of Mechanism B; error threshold of 0.025. Cellular growth and death normalized to Day 4.

Condition B (Activin A supplemented with FGF2 and BMP4) proved more difficult to describe via the investigated mechanisms, mainly because CXCR4 dynamics exhibits a faster and more prominent drop as compared to Activin A only condition. As shown in Figure 4B, the two mechanisms which give lowest error for Condition B are the ones which incorporate mesendoderm, have CXCR4 present in mesoderm and promote proliferation of all phenotypes (‘Mechanism C’) and the previously described Mechanism B. The simulated dynamics of these two mechanisms with experimental data of Condition B are shown in Figure 6. As with Condition A, although the incorporation of CXCR4 in mesoderm gives a small error, the simulated profiles of growth kinetics and cell death are not in agreement with experimental data. The next best mechanism, Mechanism B, exhibits both a low error and good results with all outputs, so it was again chosen as the most likely mechanism.

It is therefore reasonable to conclude that during endoderm induction with the conditions described above, undifferentiated stem cells first differentiate into a mesendoderm germ layer with subsequent differentiation to endoderm and mesoderm, the latter not expressing CXCR4. Furthermore, the induction condition seems to promote proliferation of both pluripotent and endoderm-like cells. The optimized parameters of this mechanism are shown in Table S2, with definitions of parameters in Table S1.

#### Model Validation

**Figure 7:**
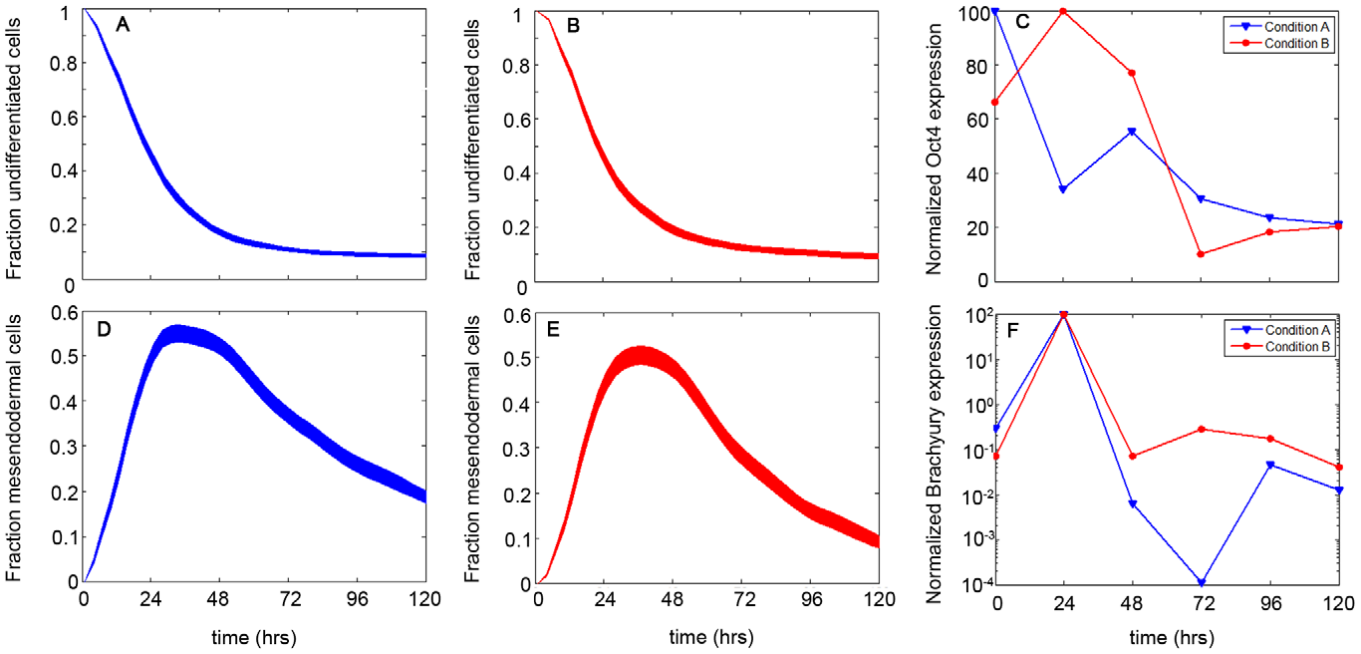
Validation of model with experimental gene expression data. Simulated dynamics of the undifferentiated (A (Condition A), B (Condition B)) and mesendoderm (D (Condition A), E (Condition B)) phenotypes were compared to experimental data of their respective genes, measured by qPCR (markers: experimental measurements; lines: linear connections between data points): Oct4 (Undifferentiated; C) and Brachyury (Mesendoderm; F). The simulated dynamics bands represent 4000 stochastic simulations using the optimized parameters of Mechanism B. mRNA levels were measured with time using qPCR. Data was first normalized to the housekeeping gene Gapdh then to undifferentiated cells. Fold change levels, determined by the 2^−ΔΔCt^ method, were then normalized to the maximum level for each respective gene (data reported as percent of maximum fold change).

The power of mathematical models lies in their predictive capacity. The predictive capacity of our proposed model was thus tested by simulating the population dynamics of cell types for which no *a priori* data was used in constructing the model. The chosen populations were that of undifferentiated cells and mesendoderm cells. The simulated profile of the undifferentiated cells (Figure 7A and B) shows an exponential decay to a final value of 10% of the cellular population. This final value was reached in approximately 3 days. The mesendoderm cell population was predicted to display more interesting dynamics, with a transient increase in cell population over the first day, followed by a decreasing trend over the next few days (Figure 7D and E).

In the next step the validity of such prediction was verified by conducting further experiments to analyze the dynamics of undifferentiated cells by Oct4 gene expression and that of mesendoderm cells by Brachyury expression. While the comparison of population dynamics with mRNA levels is not exact, under the assumption of efficient translation they become comparable. Figure 7 illustrates the comparison of experimental data with model prediction, which are found to have excellent agreement given that the model was generated with no information of these specific cellular dynamics. Oct4 levels exhibit a decay to a final value of around 20% of the maximum, at a time which correlates with simulated predictions (3 days). Brachyury levels showed a similar bimodal trend as was predicted by the model. It reached a maximum around 24 hrs, following which it gradually decayed over time.

## Discussion

The objective of the current work is to investigate the mechanism of differentiation of hESC during endoderm induction through an integrated experimental and mathematical approach. We experimentally determine the dynamics of differentiation upon endoderm induction of hESC and use these data along with a population-based stochastic model to determine the mechanisms of differentiation. The model can track growth kinetics, the dynamics of cell death, and the dynamics of differentiation into the germ layers. Thorough comparison of these simulated outputs with experimental data enables determination of the dominant mechanism of differentiation. Furthermore, the model and predicted mechanism is validated against additional experimental observations of the temporal behavior of specific cellular populations. Even though these data were not used to build the model, the model performed extremely well in capturing their dynamics.

Definitive endoderm was induced in hESC through two different pathways: the addition of Activin A and Activin A supplemented with FGF2 and BMP4. The population-based model, revised from the model originally developed for hematopoietic stem cells [13], [14], tracks individual cell behavior based on a number of set rules. The focal point of the rules is lineage propensity updating, wherein the likelihood of differentiation to a particular lineage is stochastically updated per time step. The lineages to which hESC can differentiate are definitive endoderm, visceral endoderm, and mesoderm. Depending on the specific mechanism of the model, hESC can first give rise to visceral endoderm and mesendoderm, with the latter differentiating into definitive endoderm and mesoderm. In the current model, the ectoderm germ layer has been omitted. From previous literature [24], hESC induced towards endoderm show low expression of ectoderm markers (Sox1). Commitment levels to ectoderm would be low, and therefore adding the additional ectoderm lineage would not enhance the model.

**Figure 8:**
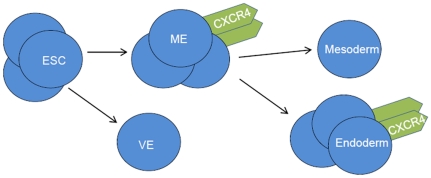
Proposed differentiation scheme of hESC during endoderm induction as generated by the population-based model. Shown is the presence of mesendoderm, the lack of CXCR4 in mesoderm, and selective phenotype proliferation. ME: mesendoderm, VE: visceral endoderm.

It is important to note that the nonlinearity observed in the differentiation dynamics contributed significantly towards identification of a robust mechanism. Sloppiness of biological parameters is well reported [23] with ranges of values being large and sensitivities between parameters varying considerably; this can make robust mechanism identification challenging. Quite interestingly, the observed dynamics of the presented study could only be explained by a single specific mechanism. Even a rigorous search of the parameter hyperspace did not yield an alternate potential mechanism. Regarding the non-linearity of CXCR4 expression, two possible explanations can be: (1) mesendoderm, expressing CXCR4, further differentiates to phenotypes which might not express the surface protein; and (2) the cellular environment might promote a higher rate of death of a certain cell phenotype which expresses CXCR4. These dynamics, along with those of Sox17, proliferation, and cell death, led us to investigate a total of 12 possible mechanisms. The majority of the mechanisms investigated was unable to capture the temporal behavior of these outputs, and therefore was discarded. The only mechanism which is able to accurately explain the experimental dynamics is one which does not have mesoderm expressing CXCR4, incorporates mesendoderm, and promotes proliferation of hESC and the mesendoderm and endoderm germ layers. This proposed mechanism is shown in Figure 8.

The endoderm induction of hESC was conducted under two different conditions with the objective of investigating mechanistic differences between these two pathways. Quite interestingly, both conditions could be explained by the same, single mechanism, while the rejected mechanisms failed to describe the dynamics even after a thorough search of the parameter space. However, there were significant differences in optimum parameter values. One prominent difference between the two conditions was their differentiation potential after being committed to the mesendoderm germ layer. ‘a0max2’ is lower for Condition B, indicating that mesendodermal cells will more quickly reach the pro-differentiation and –proliferation regimes. This is also evident from the higher level of ‘d’, although this is for both stages of differentiation. Also, cell commitment for Condition B can be considered expedited when considering the lower value of ‘xcom2’, which is the propensity threshold beyond which a mesendodermal cell is considered committed to either endoderm or mesoderm. Therefore, Activin A supplemented with FGF2 and BMP4 drives differentiation towards endoderm/mesoderm to a higher degree than Activin A alone.

As detailed earlier, the developed model for the optimum mechanism could accurately capture the experimentally observed dynamics of differentiation. Quite interestingly, only a single mechanism could adequately describe the experimental data, while the rejected mechanisms failed to describe the dynamics even after a thorough search of the parameter space.

One of the purposes of the present study was to investigate several aspects of differentiation which have faced conflicting reports in the past and to offer further insight using a mathematical analysis. One of these features is the presence of surface receptor, CXCR4. McGrath *et al*.[18] and Yusuf *et al*. [25] have reported that embryonic mesoderm expresses CXCR4 *in vivo*, depending on the stage of embryo development, whereas Takenaga *et al.* [7] reports using CXCR4 as a definitive endoderm marker with other markers used for mesoderm. Our results indicate that although both possibilities give low error with respect to Sox17 and CXCR4 population dynamics (depending on which phenotype proliferation is induced), only when CXCR4 is absent in mesoderm do we obtain qualitative agreement in the cellular growth and death temporal behavior. Furthermore, the majority of studies which follow embryo development *in vivo* or differentiation of ESC *in vitro* (for example [26]–[28]) include the mesendoderm as an intermediate phenotype arising from the differentiation of ESC which subsequently differentiates to endoderm and mesoderm rather than considering the latter two phenotypes differentiating directly from ESC. The model developed in the current study indeed comes to the same conclusion: the mesendoderm germ layer needs to be considered in order to accurately describe experimental dynamics.

## Supporting Information

Figure S1 **Implementation of mathematical model.** Pseudo-code describing flow of events in the population based model. Black: events to simulate temporal behavior of cellular population (main routine). Green: model inclusions for parameter ensemble, which runs main routine using different parameter value combinations. Red: model inclusions for sensitivity analysis, which runs main routine 4000 times (replications determined by convergence study) for each perturbed parameter value, the output being the parameter sensitivities.
(TIF)

Figure S2 **Flow cytometry data of cells positive for specific markers.** Red histogram: negative (secondary antibody only) sample. Black histogram: stained sample. Red bar is gated beyond the negative control to denote the positive sample population. (A,B): Sox17 analysis for Conditions A and B, respectively. From left to right: Day 1–4. (C,D): CXCR4 analysis for Conditions A and B, respectively. From left to right: Day 1–5.
(TIF)

Table S1 **Definitions of the parameters used in the population-based model.**
(DOC)

Table S2 **Comparison of the best fit parameter set between the two conditions.**
(DOC)
