# Supplementary figures and images for: Population Based Model of Human Embryonic Stem Cell (hESC) Differentiation during Endoderm Induction
Source: PLoS One. 2012 Mar 12;7(3):e32975. doi: 10.1371/journal.pone.0032975 (PMC3299713; doi:10.1371/journal.pone.0032975)

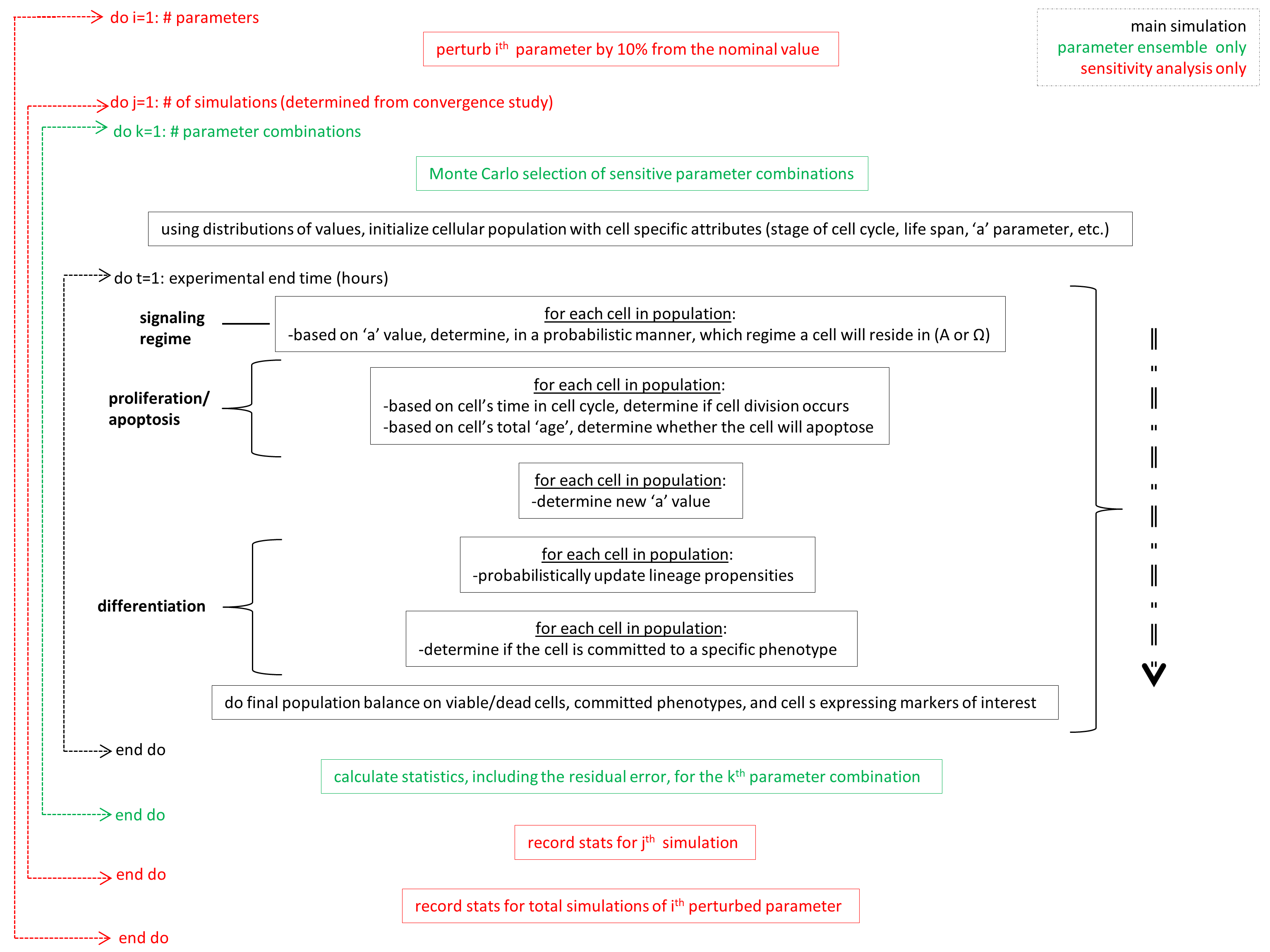

Supplement: Figure S1 — Implementation of mathematical model. Pseudo-code describing flow of events in the population based model. Black: events to simulate temporal behavior of cellular population (main routine). Green: model inclusions for parameter ensemble, which runs main routine using different parameter value combinations. Red: model inclusions for sensitivity analysis, which runs main routine 4000 times (replications determined by convergence study) for each perturbed parameter value, the output being the parameter sensitivities. (TIF) [file pone.0032975.s001.tif]

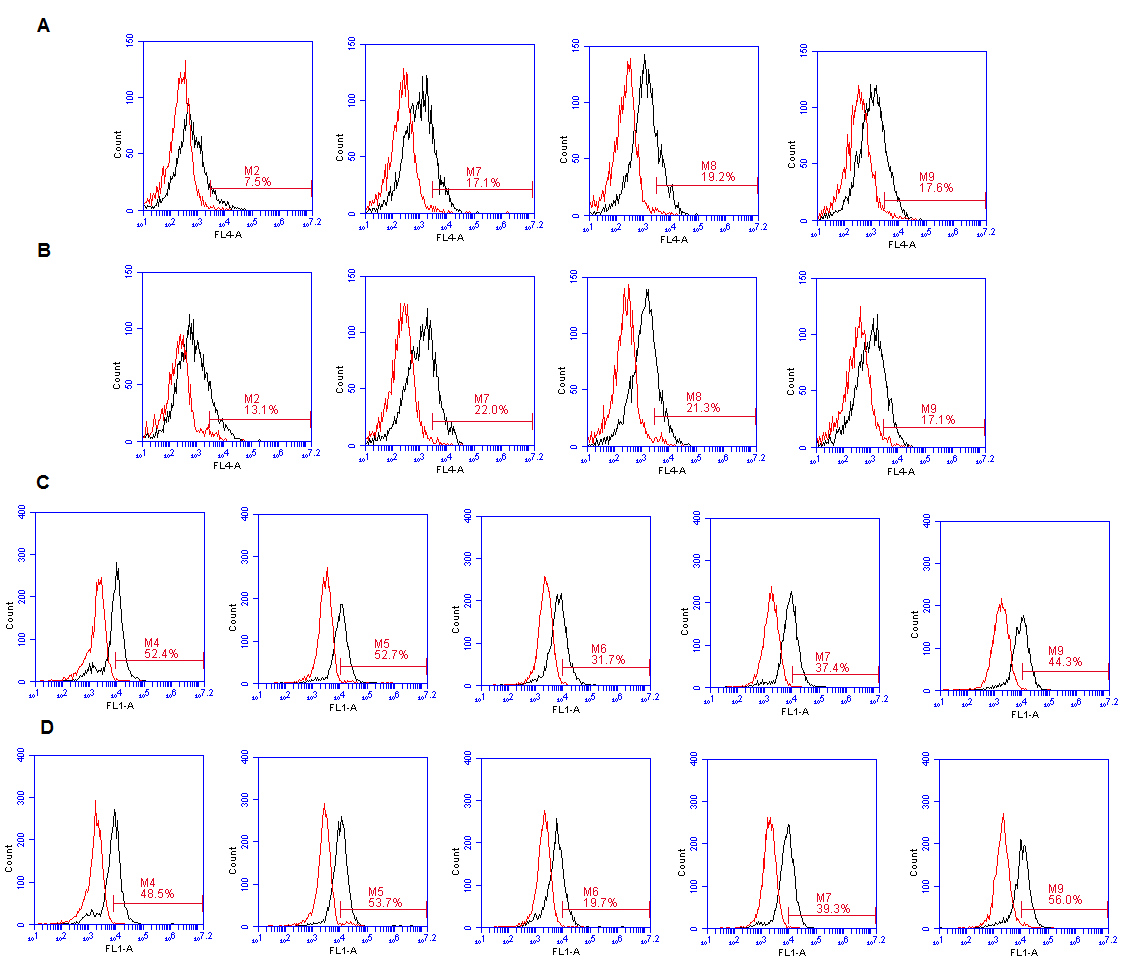

Supplement: Figure S2 — Flow cytometry data of cells positive for specific markers. Red histogram: negative (secondary antibody only) sample. Black histogram: stained sample. Red bar is gated beyond the negative control to denote the positive sample population. (A,B): Sox17 analysis for Conditions A and B, respectively. From left to right: Day 1–4. (C,D): CXCR4 analysis for Conditions A and B, respectively. From left to right: Day 1–5. (TIF) [file pone.0032975.s002.tif]
