# Supplementary material for: Population Based Model of Human Embryonic Stem Cell (hESC) Differentiation during Endoderm Induction
Source: PLoS One. 2012 Mar 12;7(3):e32975. doi: 10.1371/journal.pone.0032975 (PMC3299713; doi:10.1371/journal.pone.0032975)
Supplement: Table S1 — Definitions of the parameters used in the population-based model. (DOC) [file pone.0032975.s003.doc]

| **Parameter** | **Definition** |
| --- | --- |
| a0max | maximum value of 'a' in initial cell population, first differentiation stage |
| a0max2 | maximum value of 'a' in initial cell population, second differentiation stage |
| a0min | minimum value of 'a' in initial cell population, first differentiation stage |
| a0min2 | minimum value of 'a' in initial cell population, second differentiation stage |
| aa | used when determining the probability of a cell transferring to the alpha regime |
| amin | threshold on 'a' below which a cell is able to proliferate |
| aw | used when determining the probability of a cell transferring to the omega regime |
| ba | used when determining the probability of a cell transferring to the alpha regime |
| bprog | used in updating propensity in omega regime |
| bw | used when determining the probability of a cell transferring to the omega regime |
| ca | used when determining the probability of a cell transferring to the alpha regime |
| cw | used when determining the probability of a cell transferring to the omega regime |
| d | factor by which 'a' decreases in omega regime |
| da | used when determining the probability of a cell transferring to the alpha regime |
| dw | used when determining the probability of a cell transferring to the omega regime |
| lmax | maximum bound on cell life span |
| lmin | minimum bound on cell life span |
| nprog1^a^ | used in determining magnitude of propensity update in omega regime for lineage 1 |
| nprog2^a^ | used in determining magnitude of propensity update in omega regime for lineage 2 |
| nprog3^a^ | used in determining magnitude of propensity update in omega regime for lineage 3 |
| nprog4^a^ | used in determining magnitude of propensity update in omega regime for lineage 4 |
| nreg | used in updating propensity in alpha regime |
| tDstop | time beyond which a cell enters into a senescent stage and will not die (counted from start of cell's life) |
| tg1 | time a cell stays in the g1 phase of the cell cycle |
| tpmax | upper bound of time beyond which a cell enters into a senescent stage and will not proliferate (counted from start of cell's life) |
| tpmin | lower bound of time beyond which a cell enters into a senescent stage and will not proliferate (counted from start of cell's life) |
| xcom | threshold level of propensity beyond which a cell is considered committed, first differentiation stage |
| xcom2 | threshold level of propensity beyond which a cell is considered committed, second differentiation stage |

^a^For the mechanisms which include mesendoderm: lineage 1-mesendoderm, lineage 2-visceral endoderm, lineage 3-definitive endoderm, lineage 4-mesoderm. For the mechanisms which exclude mesendoderm: lineage 1-definitive endoderm, lineage 2-mesoderm, lineage 3, visceral endoderm
