# Supplementary material for: Population Based Model of Human Embryonic Stem Cell (hESC) Differentiation during Endoderm Induction
Source: PLoS One. 2012 Mar 12;7(3):e32975. doi: 10.1371/journal.pone.0032975 (PMC3299713; doi:10.1371/journal.pone.0032975)
Supplement: Table S2 — Comparison of the best fit parameter set between the two conditions. (DOC) [file pone.0032975.s004.doc]

| **Parameter** | **Condition A** | **Condition B** |
| --- | --- | --- |
| a0max2 | 0.3 | 0.161 |
| xcom | 0.8 | 0.767 |
| xcom2 | 0.9 | 0.79 |
| d | 1.2 | 1.32 |
| tg1 | 12 | 14.6 |
| lmax | 190 | 222 |
| nprog(ME) | 0.11 | 0.0879 |
| nprog(endoderm) | 0.04 | 0.0278 |
| nprog(mesoderm) | 0.06 | 0.0457 |
| nprog(VE) | 0.06 | 0.0611 |
| aa | 2 | 2.07 |

Only the sensitive parameters are shown. A ‘2’ after the parameter denotes the parameter for the second stage of differentiation (mesendoderm to mesoderm and endoderm) as opposed to the first stage (hESC to mesendoderm and visceral endoderm). ME: mesendoderm, VE: visceral endoderm.
